# Analyzing 7000 texts on deep brain stimulation: what do they tell us?

**DOI:** 10.3389/fnint.2015.00052

**Published:** 2015-10-26

**Authors:** Christian Ineichen, Markus Christen

**Affiliations:** ^1^Institute of Biomedical Ethics and History of Medicine, University of ZurichZurich, Switzerland; ^2^Preclinical Laboratory for Translational Research into Affective Disorders, Clinic for Affective Disorders and General Psychiatry, Psychiatric University Hospital ZurichZurich, Switzerland; ^3^University Research Priority Program Ethics, University of ZurichZurich, Switzerland

**Keywords:** deep brain stimulation, text analysis, network analysis, co-occurrence of terms, bibliometrics, bioethics

## Abstract

The enormous increase in numbers of scientific publications in the last decades requires quantitative methods for obtaining a better understanding of topics and developments in various fields. In this exploratory study, we investigate the emergence, trends, and connections of topics within the whole text corpus of the deep brain stimulation (DBS) literature based on more than 7000 papers (title and abstracts) published between 1991 to 2014 using a network approach. Taking the co-occurrence of basic terms that represent important topics within DBS as starting point, we outline the statistics of interconnections between DBS indications, anatomical targets, positive, and negative effects, as well as methodological, technological, and economic issues. This quantitative approach confirms known trends within the literature (e.g., regarding the emergence of psychiatric indications). The data also reflect an increased discussion about complex issues such as personality connected tightly to the ethical context, as well as an apparent focus on depression as important DBS indication, where the co-occurrence of terms related to negative effects is low both for the indication as well as the related anatomical targets. We also discuss consequences of the analysis from a bioethical perspective, i.e., how such a quantitative analysis could uncover hidden subject matters that have ethical relevance. For example, we find that hardware-related issues in DBS are far more robustly connected to an ethical context compared to impulsivity, concrete side-effects or death/suicide. Our contribution also outlines the methodology of quantitative text analysis that combines statistical approaches with expert knowledge. It thus serves as an example how innovative quantitative tools can be made useful for gaining a better understanding in the field of DBS.

## Introduction

A characteristic of modern knowledge production is the enormous increase of the number of scientific publications (original papers, reviews, conference abstracts, editorial material, etc.) that is made accessible through digital technology. In neuroscience alone, it is estimated that more than 100,000 papers a year are added to a text corpus that contains many millions of publications (Grillner, 2014). This information overload poses a substantial challenge for researchers to keep pace with the developments in their own fields; and it is well-known that the biomedical sciences are especially vulnerable in this regard, since they are strongly oriented toward text-based knowledge sources (Hölzer et al., 2006). This problem certainly also holds within the field of Deep Brain Stimulation (DBS) (Hariz et al., 2013), that has experienced a substantial growth of publications since the late 1990s (Müller and Christen, 2011). In this paper we propose a way to handle this challenge by using quantitative text analysis that combines statistical approaches with expert knowledge.

Quantitative approaches using bibliometrics, scientometrics, and text mining have gained popularity, as they may serve as navigational prospects and orientation aids. They enable researchers to identify relevant topics, trends, and publications in a fast-growing text corpus. Among other methods, network approaches, and data visualization techniques that aim to identify connections between topics within a given text corpus are being used (Popping, 2000, 2003; Ryan, 2007) and have shown to be useful to grasp important concepts within a text of any length. While being applied in a wide field, such approaches have a long tradition in enabling researchers exploring possible configurations of the unknown, shared visual representations which may open new ways for channeling collective attention, envisaging innovative interpretations and help us to make sense of data at different scales (Okada et al., 2014). The ultimate advantage of network analyses and their visual representation in general is recognized from a wide and diverse field. Ideally, the results of such a methodological approach will verify conjectured trends within the field, enrich the discourse, and support unconventional ideas or interpretations of the ongoing scientific development. In the following, we will explore the techniques of sized graphs in combination with sophisticated text preprocessing in order to find features in the network structure of the DBS text corpus which otherwise would be difficult to detect.

In general, a graph visualizes relations of a given set of data and is composed of nodes and edges. Nodes typically represent items or concepts whereas edges connect nodes according to some association rules. Graphs are widely used for e.g., analyzing social networks where people represent the nodes and edges represent relationships between people. To convert information (e.g., of text) into a visual representation can facilitate the handling and perception of hidden structures from large data sets. By following paths and detecting clusters of closely related nodes, one may detect unique features of a given data set. However, if the data set exceeds a certain level of magnitude, the task of exploring, and navigating becomes increasingly difficult. More specifically, there is extensive work on representing textual data as graphs and the subsequent application of network text analysis (e.g., Losiewicz et al., 2000; Grbic et al., 2013; James et al., 2013; Guan et al., 2014) for gaining an increased understanding of influential concepts, text's meanings, and structure. Network analysis is therefore also suitable for linguistic comparative analyses which focus on semantic relations between words, often framed through the co-occurrence of terms (i.e., relevant terms that more often appear in the same text are more likely to share some semantic connection). By making use of the large number of published DBS papers as well as a statistics driven quantitative approach, a potential subjective bias may be diminished.

In the following, we will use graph analysis and visualization techniques for investigating (1) most influential topics, (2) their mutual connections as well as (3) the temporal development of topics by retrieving the titles and abstracts of all published publications from 1991 to 2014 in the field of DBS (see Material and Methods for more specific information). We expect to be able to reproduce known phenomena (e.g., an increase in discussing psychiatric disorders in the DBS literature, well-described anatomical targets for the treatment of various disorders, or known treatment methods for various disorders) which might become obvious in different ways (e.g., direct connections or by reference to how e.g., anatomical targets are being discussed). Additively, we are interested in detecting how specific topics (e.g., lesioning-methods, personality, and bioethics) develop over time and/or how they interrelate with other topics. The original text corpus was composed of more than 10,000 DBS publications, based on which 7154 texts (titles and abstracts) containing more than 400,000 potentially relevant words have been selected for analysis. Using the co-occurrence of key terms as association rule, we conducted graph visualization techniques, community analyses and quantitative metrics to get insight into how DBS has been discussed during the last 23 years.

The results of this analysis are then reflected by referring to issues that dominated the DBS literature. Beside others, we are interested in how some topics that have been identified as ethical focal points in the international practice of DBS (Christen et al., 2014) are represented in this quantitative approach. In this way we explore the potential of such quantitative approaches for identifying subject matters that are of relevance from a bioethical perspective. The study will conclude by a discussion of limitations of quantitative approaches as heuristics to deal with information overload.

## Material and methods

Textual data is complex due to syntactic (verb forms, declination, etc.), semantic (homonymy, synonymy, etc.) and pragmatic (context-dependency etc.) variation. Therefore, any quantitative analysis based on textual data has to ensure appropriate preprocessing of text data such that it can be correctly used for statistical processing. In the following, we first describe text preprocessing to generate the final word set that was then used for trend and co-occurrence analysis, before we outline the network analysis and visualization methodology. The aim of the study was to obtain a comprehensive set of DBS publications as a set for quantitative analysis. We restricted ourselves to papers published since 1991, as earlier papers on DBS are rather sparse and do not yet contain in all cases the string “deep brain stimulation” as a simple identifier for a text that can be attributed to the DBS text corpus.

### Text preprocessing

**Figure 1:**
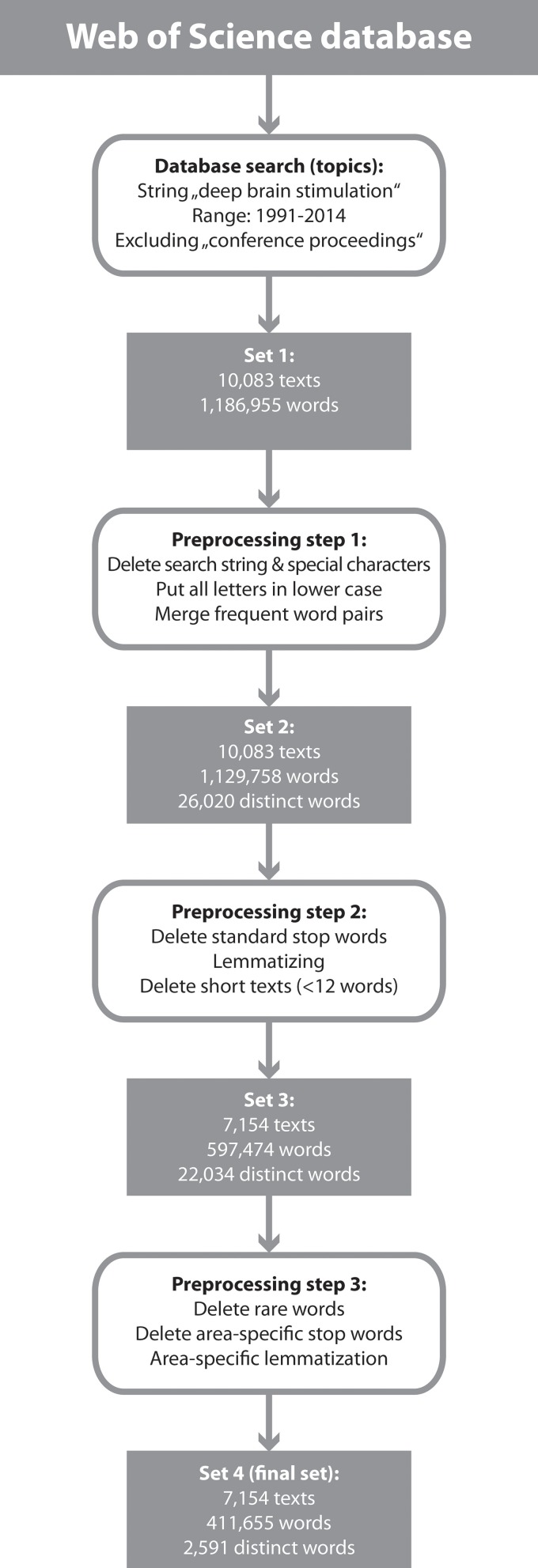
**Scheme outlining the generation of the final text set for network analysis**.

Text preprocessing contained three steps as outlined in Figure 1. The starting point was a search in the Web of Science Core Collection database (the search was performed on December 5th 2014)^1^. We used the search string “deep brain stimulation” in the “topics”-field, restricted to the time range 1991 to 2015. We excluded the “Proceedings Citation Index,” because entries in this database only contain the title of contributions without abstract. This resulted in a set of more than 10,000 contributions and a text corpus of almost 1.2 million words. In a first preprocessing step, we deleted special characters (e.g., “(” or “?” including number signs) as well as the search string itself (because it is unspecific), we transformed all letters into lower case and we merged frequent word pairs (e.g., “informed consent” to “informedconsent”). This last step was based on a word-pair statistics over the whole text set to identify very frequent pairing of words (a cutoff value of about 80% was chosen). We identified frequent word pairs by selectively looking through potential word-pairs and decided whether they should be merged based on our experience with the DBS vocabulary (in total, 130 word pairs were merged).

In a second preprocessing step, we deleted standard stop words^2^ like “the,” “is” etc., and we performed a lemmatization, i.e., we transformed all nouns, verbs, and adjectives into their ground form using standard lookup tables^3^; for example the plural “brains” is replaced by “brain” or the past tense “came” is replaced by “come”—the latter step served for removing the amount of variability. We refrained from stemming (another standard procedure in text processing), because a stemmer operates on a single word without knowledge of the context, and therefore cannot discriminate between words which have different meanings depending on the text. Finally, we computed the text length distribution and we deleted all short texts^4^. The remaining text corpus consisted of 7154 texts and 597,474 words, 22,034 of which were distinct words.

Finally, a third preprocessing step was necessary due to area-specific stop-words and terms that were not contained in standard lemmatizing lists. We first deleted all words that were present in less than 0.1% of the texts (i.e., that are contained in maximal seven texts), because these rare words are not suitable for statistical text analysis. Then, two raters (the authors) independently assessed which words are considered to be unspecific. If both raters independently rated the same words as unspecific, they were deleted (1308 in total). In a similar way, we identified 1380 replacement-pairs for area-specific lemmatization^5^. In this way, we generated a set of 7154 texts that consisted of 411,655 words, consisting of 2591 distinct words. This set was used for the quantitative analysis.

**Figure 2:**
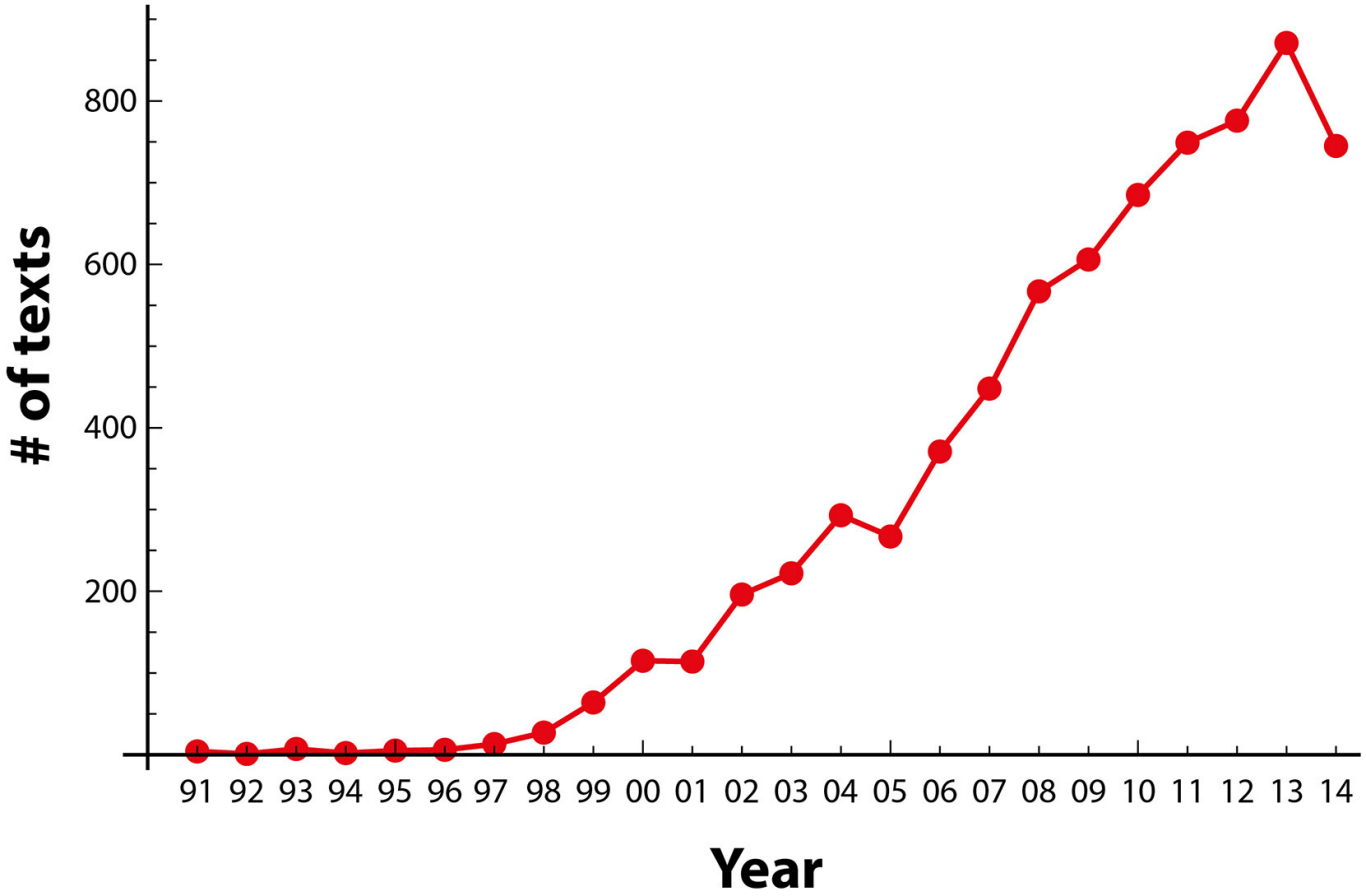
**Distribution of publication years of all texts of the final set (7154 texts), indicating a steep growth in the number of DBS texts since the late 1990s**.

Figure 2 provides an overview of the text corpus in terms of publication years which shows the quantitative basis for our analysis and reflects the substantial growth of publication within the field of DBS. Text size distribution and term frequency are shown in Supplementary Figure 1.

### Text analysis

The text analysis consisted of an expert evaluation part and a statistical part. The expert evaluation (performed by the authors) aimed to identify terms that are characteristic for issues and topics that are widely discussed in the field of DBS. For issue identification, we also referred to earlier publications from us, i.e., we included issues and topics which were identified as relevant based on an analysis of DBS conference contributions (Christen and Müller, 2011) and a large review covering the literature on DBS in the Nucleus subthalamicus (Christen et al., 2012). “Issues” refer to overarching themes (such as anatomical localization), “topics” refer to defined subject matters within an issue (such as specified anatomical localizations), and “terms” refer to the actual words that appear in the text. As outlined in Table 1, seven issues containing 73 topics were chosen for further analysis, based on the authors estimation of relevance (some topics could be in more than one issue; e.g., pain as indication or side-effect). Topics sometimes are composed of different terms (e.g., “accumbens” and “nucleusaccumbens”). In that case, the terms describe the same topic.

**Table 1:** **Issues and associated topics, characterized by terms that were contained in the final set**.

**Issue**	**Topics associated with issue (in brackets: terms that characterize the topic)**
Anatomical localization	15 topics:
	{accumbens, nucleusaccumbens}, {alic, limb}, {amygdala}, {caudatenucleus}, {centromedian, centromedianparafascicularcomplex}, {cingulatecortex}, {cingulum}, {globuspallidus, globuspallidusexternus, globuspallidusinternus, pallidus}, {hippocampus}, {pallidum}, {pedunculopontine, pedunculopontinenucleus}, {stn, subthalamicus}, {subgenual, subgenualcingulate, subgenualcingulatecortex}, {vim}, {zonaincerta}
DBS indication	20 topics:
	{addiction, alcoholism, smoke}, {alzheimer}, {anorexianervosa, eatingdisorders, obesity}, {anxiety}, {ataxia}, {bradykinesia}, {chorea, huntington}, {clusterheadache, headache}, {depression}, {dyskinesia}, {dystonia}, {epilepsy}, {essentialtremor, tremor}, {hypomania, mania}, {memory}, {obsessivecompulsive, ocd}, {parkinson}, {schizophrenia}, {sclerosis}, {tourette}
Positive effects	3 topics:
	{qualityoflife, wellbeing}, {alleviation, relief, remission}, {enhancement}
Negative effects	18 topics:
	{safety}, {aberrant, adverse, adverseevents, complication, decline, deterioration, distress, impairment, perseverative, sequela, sideeffect}, {apathy}, {ataxia}, {character, personality}, {death, die, suicide}, {dysarthria}, {dyskinesia}, {fluency, language, speech}, {hemorrhage, hemorrhage}, {hypersexuality}, {hypomania, mania}, {impulsecontrol, impulsivity}, {infection, inflammation}, {memory}, {pain}, {psychosis}, {psychosocial}
Methodological issues	15 topics:
	{ablation}, {radiosurgery, ultrasound, gammaknife}, {capsulotomy}, {cingulotomy}, {pallidotomy}, {subthalamotomy}, {thalamotomy}, {psychosurgery, lobotomy, leucotomy}, {computertomography, diffusiontensorimaging, eegfmri, electrocorticography, electroencephalography, fmri, magnetoencephalography, mri, tomography, transcranialsonography, ventriculography, pet, spect}, {spinalcordstimulation}, {transcranialdirectcurrentstimulation}, {transcranialmagneticstimulation}, {vagusnervestimulation}, {electroconvulsivetherapy}, {dopamine, dopaminereplacementtherapy, duodopa, ldopa}
Economic issues	3 topics:
	{commercial, cost, costeffectiveness, economic, expensive, financial, inexpensive, market, socioeconomic, expenditure}, {industry, manufacture, manufacturer, medtronic, kinetra}, {effectiveness}
Technological issues	3 topics:
	{battery, cable, device, electrode, hardware, implantablepulsegenerator, lead, pacemaker, recharge, rechargeable, stimulator, wireless}, {closedloop, responsiveneurostimulatorsystem}, {program}

*Remind that Lemmatizing in step 3 has mapped most of the different manifestations of topics (e.g., abbreviations) on a single term (e.g., the abbreviation “PD” on “parkinson”). The topics ataxia, dyskinesia, (hypo)mania, and memory are present in two issue classes*.

In addition, we analyzed ethical issues as a single topic characterized by the terms “ethic,” “moral” and “social” because we were interested in investigating on how ethical aspects are being discussed in the literature. In total, we had 74 topics and 162 terms. For these topics, we performed a trend analysis, i.e., we counted the appearance of terms belonging to different topics in all texts of a single year starting from 2000 (due to the low number of texts in the 1990s that contained topic terms). We always normalized the trend data with the total number of publications per year for detecting trends within the whole DBS publication body.

Furthermore, we calculated the pairwise *co-occurrence C*(*X, Y*) of two topics *X* and *Y* as:

$$\begin{array}{l} C\left(X,Y\right)=\frac{\left|T\left(X,Y\right)\right|}{\text{min}\left(\left\{\left|X|,|Y\right|\right\}\right)} \end{array}$$

Where |*T*(*X, Y*)| denotes, how often the terms characteristic for topic *X* and *Y* appear in the same text and |*X*| respectively |*Y*| denotes, in how many texts these terms appear in the whole set. *C*(*X, Y*) is between 0 (the terms of two topics never occur in a same text) and 1 (the terms always occur in same texts). The co-occurrence is used as similarity metrics for the network analysis.

For visualizing the co-occurrence matrix, we used Gephi, an open source software for analyzing graphs and networks^6^. In the resulting graph, the thickness of the edges reflects the co-occurrence, i.e., a higher probability that two terms appear in the same text is reflected by a thicker and more saturated connection.

The sizes of the nodes (= topics) reflect their betweenness centrality (BC), which is equal to the number of shortest paths from all vertices to all others that pass through that node. The betweenness centrality *BC*(*X*) of topic *X* is defined as:

$$\begin{array}{l} BC\left(X\right)=\underset{X\neq Y\neq Z}{\sum }\frac{\sigma_{Y,Z}\left(X\right)}{\sigma_{Y,Z}} \end{array}$$

Where σ_*Y, Z*_ is the number of shortest paths between topics *Y* and *Z*, and σ_*Y, Z*_(*X*) is the number of shortest path between those two topics that pass through *X*. For example, if there are three different shortest paths between two nodes and a third node is part of two of them, then the BC of this third node and for this specific configuration is 2/3. As a result, nodes with higher BC are more influential, because they functions as junctions for “communication” within the network (Freeman, 1977; Brandes, 2001). Terms with high BC are therefore hypothesized to play the most important role in establishing the meaning for the text and its interpretation.

We visualized the whole network of all topics as well as the networks that only contained topics of two issue classes (we performed six specific visualizations in total: anatomical targets-indication, indication-side effects, anatomical targets-side effects, technological issues-indication, economic issues-indications, positive-effects-anatomical targets. In the following, we display the three of them that yielded the most interesting results.

In some cases, we also looked at Page rank values of each node in order to make a statement about the importance of the term. Page rank is an algorithm that was originally developed to measure the relative importance of web pages. It formed the basis for ranking results when using the Google search engine and was named after Larry Page (Brin and Page, 1988; Page et al., 1999). Today, Page rank is a common tool in network analysis aiming at assessing linked documents based on their connectivity structure. The principle of this measure can be explained as follows: the more links (in our case connections) refer to a site (in our case to a node/topic), the more weight a given site receives. As a consequence, the more weight a given site/node acquires, the bigger its importance. If one interprets co-occurrence of topics as a measure of “linking” two topics, then the page rank value would determine the order of “search results” in the network determined by the topics chosen by us. Since we are only rarely referring to page rank, we refrain from describing the mathematical basis of this algorithm in detail and refer to the original work by Brin and Page, to a brief description by Chen et al. (2007 p. 9) and to an in-depth review by Langville and Meyer (Brin and Page, 1988; Langville and Meyer, 2004; Chen et al., 2007).

Graphs were represented by use of a Force Atlas 2 algorithm (Jacomy et al., 2014). This algorithm is used to spatialize the network: nodes repulse each other similar to charged particles whereas edges attract their nodes like springs. The specific spatial distribution of each node therefore depends on the nodes' connections to other nodes. As a result, the specific coordinate of one single node cannot be interpreted on its own but has to be analyzed in combination with other nodes (Jacomy et al., 2014). Since edges are weighted, we added the “Edge Weight Influence” δ (δ = 3.0, a pre-programmed selection option) to the visualization in order to prevent edge weights to be ignored.

## Results

### Trend analysis over time

**Figure 3:**
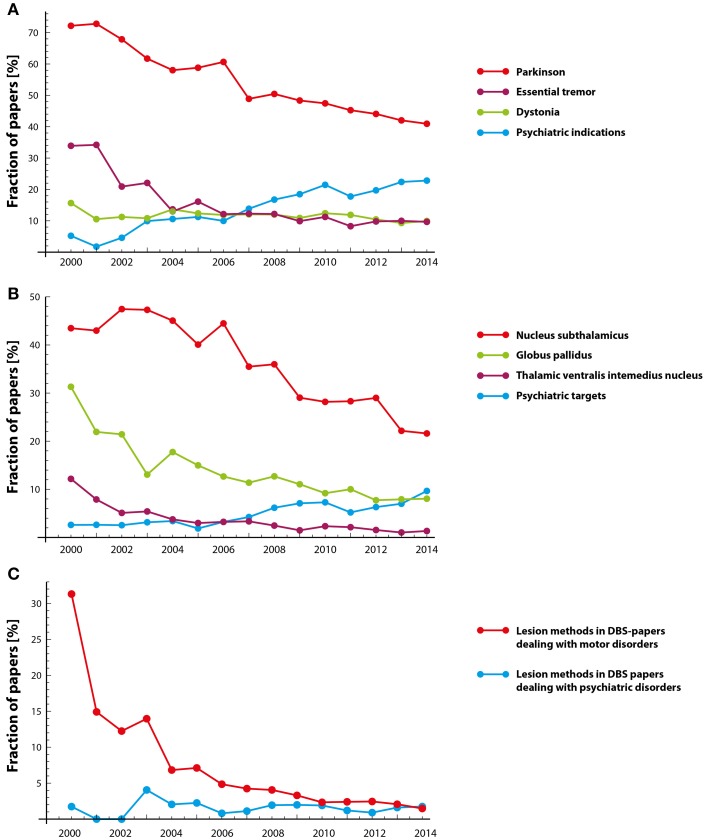
**Trend analysis for selected topics. (A)** Movement disorders vs. psychiatric indications; **(B)** DBS targets for movement disorders vs. targets for psychiatric indications; **(C)** mentioning of lesion methods in papers also mentioning movement disorders vs. psychiatric indications.

The trend analysis of potential anatomical DBS targets over time suggests a crosscurrent tendency: while the discussion of psychiatric DBS indications such as addiction, major depressive disorder (MDD), schizophrenia, Tourette syndrome (TS), and obsessive-compulsive disorder (OCD) (among others) are increasingly being discussed, the discussion of conventional, motor-related indications such as Parkinson's disease (PD) and essential tremor (ET) recedes (see Figure 3A). Dystonia, on the other hand, shows a surprisingly stable pattern over time. In confirmation of the above, the trends for anatomical DBS targets mainly match the ones depicted in the DBS indication analysis: while traditional anatomical targets used in movement disorder therapy decline over time—globus pallidus (GP), ventral intermediate nucleus (Vim), subthalamic nucleus (STN) –, a marked increase of “psychiatric” targets—e.g., nucleus accumbens (Nacc) or subgenual cingulate (SG)—is visible (see Figure 3B). Interestingly, the increase of psychiatric targets is less pronounced than the one for psychiatric indications, suggesting that psychiatric indications have become *per se* an emerging topic within the DBS literature. The above described pattern is again partly backed when including the trend analysis for lesion methods (including radiosurgery, capsulotomy, gammaknife, pallidotomy, to name a few) specifically: the discussion of such alternative techniques in the context of motor disorders decreases substantially. Interestingly, there is no trend in the case of psychiatric indications (see Figure 3C).

In line with these results is the fact that overall, the trend analysis for negative effects shows an increased emphasis in discussing “psychiatric” phenomena (including anhedonia, hypomania, personality, and impulsivity among others) whereas phenomena associated with traditional, motor related indications (apraxia, ataxia, dysarthria, among others) are consistently less often discussed. Surgery related issues (such as hemorrhage, infection, and ischemia, to name a few) are quite stable. The data also confirm earlier findings (Müller and Christen, 2011) that general terms which indicate side-effects non-specifically such as “aberrant,” “adverse,” “complication,” “distress,” “impairment,” “sequel,” “sideffect” (among others), are also less often mentioned.

Finally, technological terms associated with new stimulator systems such as “closed loop,” “responsive neurostimulator system,” “rechargeable,” and “wireless” have partly displaced the discussion about conventional technological and hardware-related terms which themselves are less often discussed (e.g., “battery,” “cable,” “electrode,” “implantable pulse generator,” “lead,” “pacemaker,” and “stimulator”; data not shown).

### Analysis of whole data set

#### General structure of the graph

**Figure 4:**
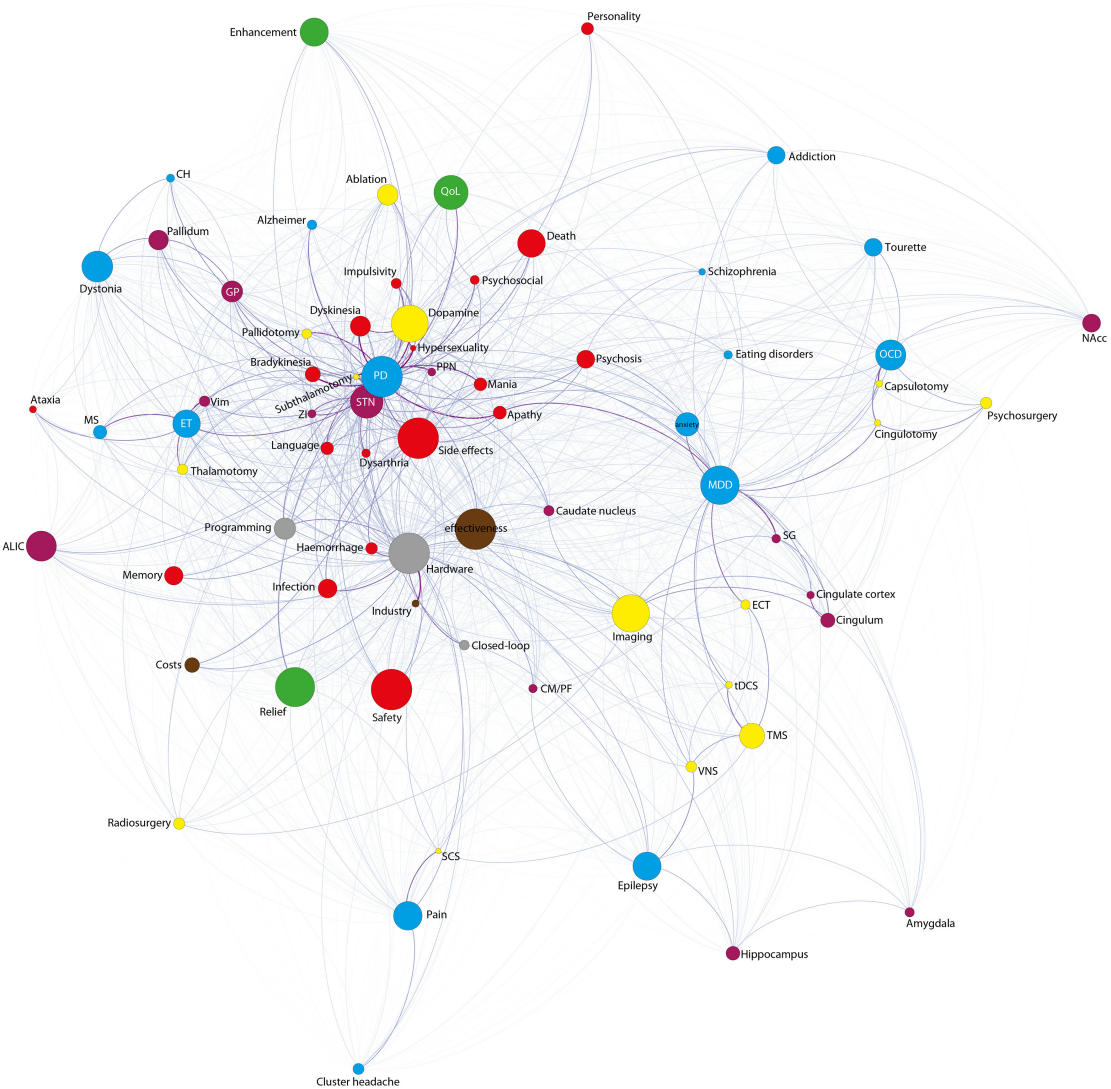
**Network of all 73 topics (except ethic)**. Color-code: Blue: indications; purple: anatomical targets; yellow: methods; green: positive effects; red: negative effects; brown: economic issues; gray: technological issues.

Overall, the aggregated graph (except the issue ethic which was analyzed separately) consists of 73 nodes and 1908 edges (Figure 4). We observe the average shortest path length to be pretty small (1.3) (Jackson, 2008) (i.e., we can move from one point in the network to another point quite easily, the graph is therefore well-interconnected). The graph furthermore shows a high number of influential nodes besides a high value of average degree (i.e., a quite diverse text corpus). In general, degree is a measurement of connectedness in graph theory which means that a specific node in a given network with high degree consequently has many neighbors in that network. A high average degree therefore means that the graph is highly interconnected. Because of this high average degree, no contextual clusters have been identified using the community detection algorithm of Blondel et al. (2008).

#### Thematic structure of DBS publications

The betweenness centrality (BC) analysis reveals that apparently five main topics dominated the DBS field in terms that they occupied an exceedingly central space within the whole text corpus. Those are effectiveness, safety, side-effects, and hardware related issues apart from PD, the main indication for DBS (equal BC values; see Figure 4 and Table 1 in Supplementary Materials). Moreover, those topics are adjacent to most of the words in the network and therefore function both as local hubs (i.e., a node with many connections) and as important junctions within the whole text corpus.

Apart from the five main topics, the topics including positive effects (alleviation, relief, remission), MDD, imaging methods, dopamine, quality of life (QoL), STN, dystonia, OCD, anterior limb of the internal capsule (ALIC), pain, enhance(ment), epilepsy, death, ET, and imaging methods (with decreasing values across sequence) also show high betweenness centrality. Concrete side-effects appear at place 25 and 27 (dyskinesia and infection). The topics personality (place 41), psychosocial (place 56)—both inherently difficult variables –, subthalamotomy, alternative therapies [such as electro-convulsive therapy (ECT), vagus nerve stimulation (VNS), spinal cord stimulation (SCS), or transcranial direct current stimulation (tDCS)] and hypersexuality [which itself occurs quite rarely (in 19 abstracts only)] (place 72) receive especially low BC.

A Google's page rank analysis revealed the following sequence of importance: PD, hardware related terms, side-effect, STN, MDD, effectiveness, dopamine, ET, imaging-methods, GP, OCD, safety, dystonia, and positive effects (alleviation, relief, remission). The BC values of neuroanatomical targets indicates a higher BC value for ALIC than for GP and a higher one for Nacc than for Vim. However, this sequence changes when conducting Page-rank analysis: highest values are accredited to the STN, GP, ALIC, Vim, and Nacc. Also, the lesional approaches (pallidotomy, subthalamotomy, and thalamotomy) receive dramatically more weight (in the middle field) when performing Page-rank (among lowest if conducting BC).

### Analysis of specific topics

Next we performed a co-occurrence analysis incorporating the five terms (effectiveness, safety, side-effect, PD, and hardware) with highest BC (see Table 2 in Supplementary Materials for detailed information on all co-occurrence-values). Firstly, the topic effectiveness is most often mentioned in combination with the topics PD and hardware. Likewise, the second topic safety is most often discussed in the context of PD and hardware. Third, we were interested in the topic side-effect, which shows to be, apart from PD, the topic with most above-threshold co-occurrences (co-occurrences > 0.3, determined by the authors based on distribution of the data; see Table 2 in Supplementary Materials). It is most often mentioned with hardware- and motor-related side-effects but also includes side effects of the psychiatric/psychological domain: infection, hemorrhage, dysarthria, apathy, speech, psychosis, memory, mania, dyskinesia, psychosocial, anxiety, hypersexuality, subthalamotomy, STN (among others, with decreasing values across sequence). Fourth, the main indication PD, is discussed to a greater extent with hypersexuality (or more accurately; whenever there is a text including the term “hypersexuality,” “parkinson” is most often also present), bradykinesia, subthalamotomy, and apathy. Please note that the term “hypersexuality” appears quite rarely (in 19 abstracts only). The results therefore have to be complemented and analyzed in combination with how often a term actually occurs within the texts (for frequency distributions see Supplementary Figure 1B). The last and fifth key-topic with highest BC includes hardware-related issues which is discussed most often with industry, hemorrhage, new systems (terms: “closed loop,” “responsive neurostimulation system”), program, infection, PD, and general economic-topics (terms “cost,” “commercial,” “economic,” “financial” among others).

### Analysis of interactions between different issues

In order to investigate on potential interactions of different issues, we conducted a co-occurrence analysis. First we outline the co-occurrence of topics related to the issues indications, anatomical targets, and side-effects.

#### Interaction between indications and side-effects

**Figure 5:**
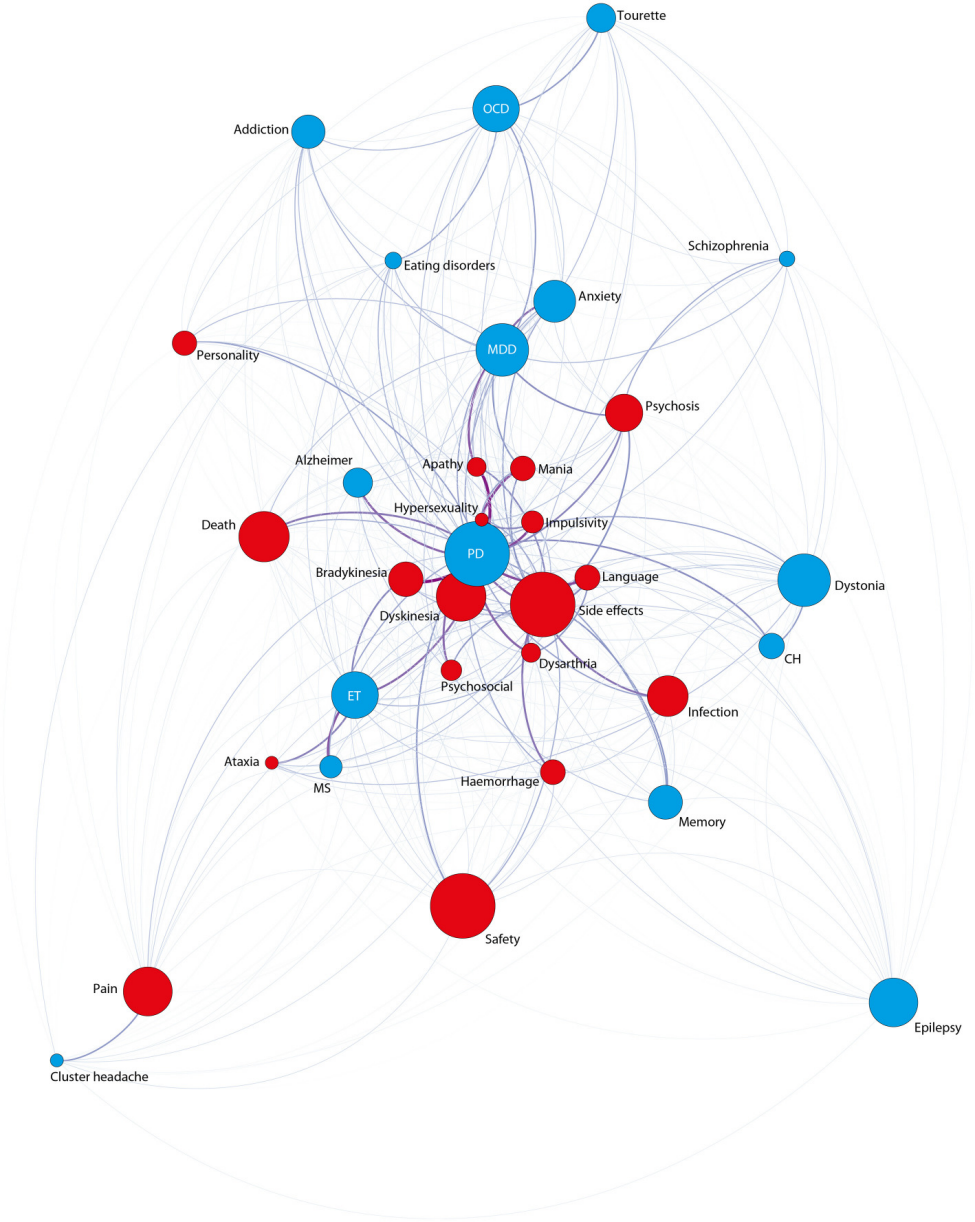
**Network-Analysis for the issues indications (blue nodes) and side-effects (red nodes)**.

As for the combination of indications and side-effects (Figure 5), the strongest co-occurrence connections yielded the following results. The topic PD is most commonly discussed with hypersexuality (as already stated above). Moreover, PD is often discussed with apathy, dyskinesia, mania, impulsivity, speech (and dysarthria), psychosocial, death/suicide, and psychosis. ET, on the other hand, is most often discussed with dysarthria. Of note is the fact that neither MDD nor dystonia is strongly connected to any concrete side-effect. Additionally, and as a side note, an intracategorial analysis shows most often co-occurring side effects to be impulsivity and hypersexuality.

#### Interaction between indications and anatomical targets

**Figure 6:**
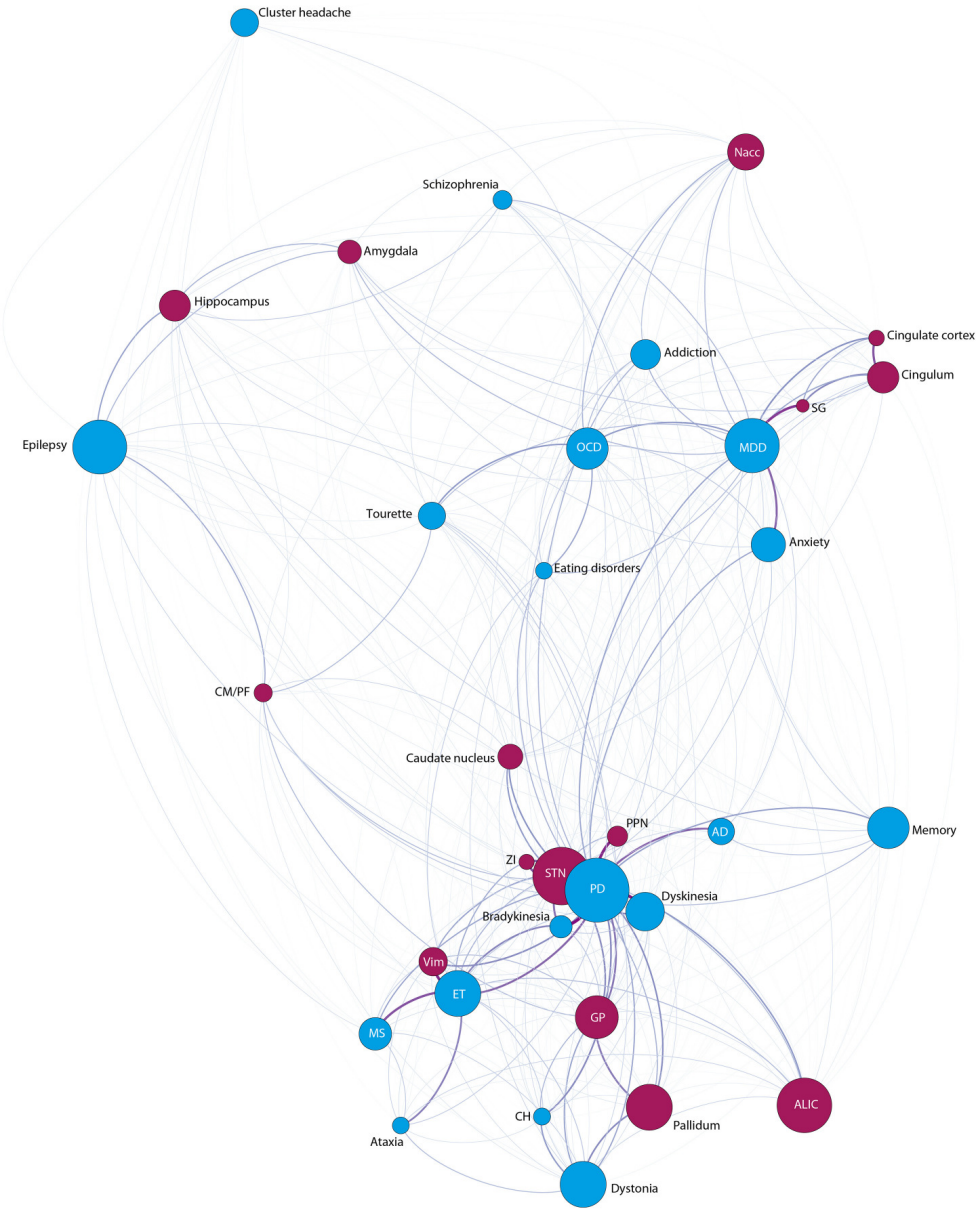
**Network-Analysis for the issues indications (blue nodes) and anatomical targets (purple nodes)**.

The combination of indications and targets (Figure 6) showed strongest co-occurrence connections between PD and the STN, followed by the pedunculo-pontine nucleus (PPN), the zona incerta (ZI) and lastly with the GP. ET, on the other hand, is clearly connected with the Vim. MDD is most often linked to the SG, the cingulate cortex, and Nacc, while the indications bradykinesia and dyskinesia show most frequent connections to the STN. Finally, OCD is most often discussed with the Nacc and schizophrenia with the hippocampus.

#### Interaction between side-effects and anatomical targets

**Figure 7:**
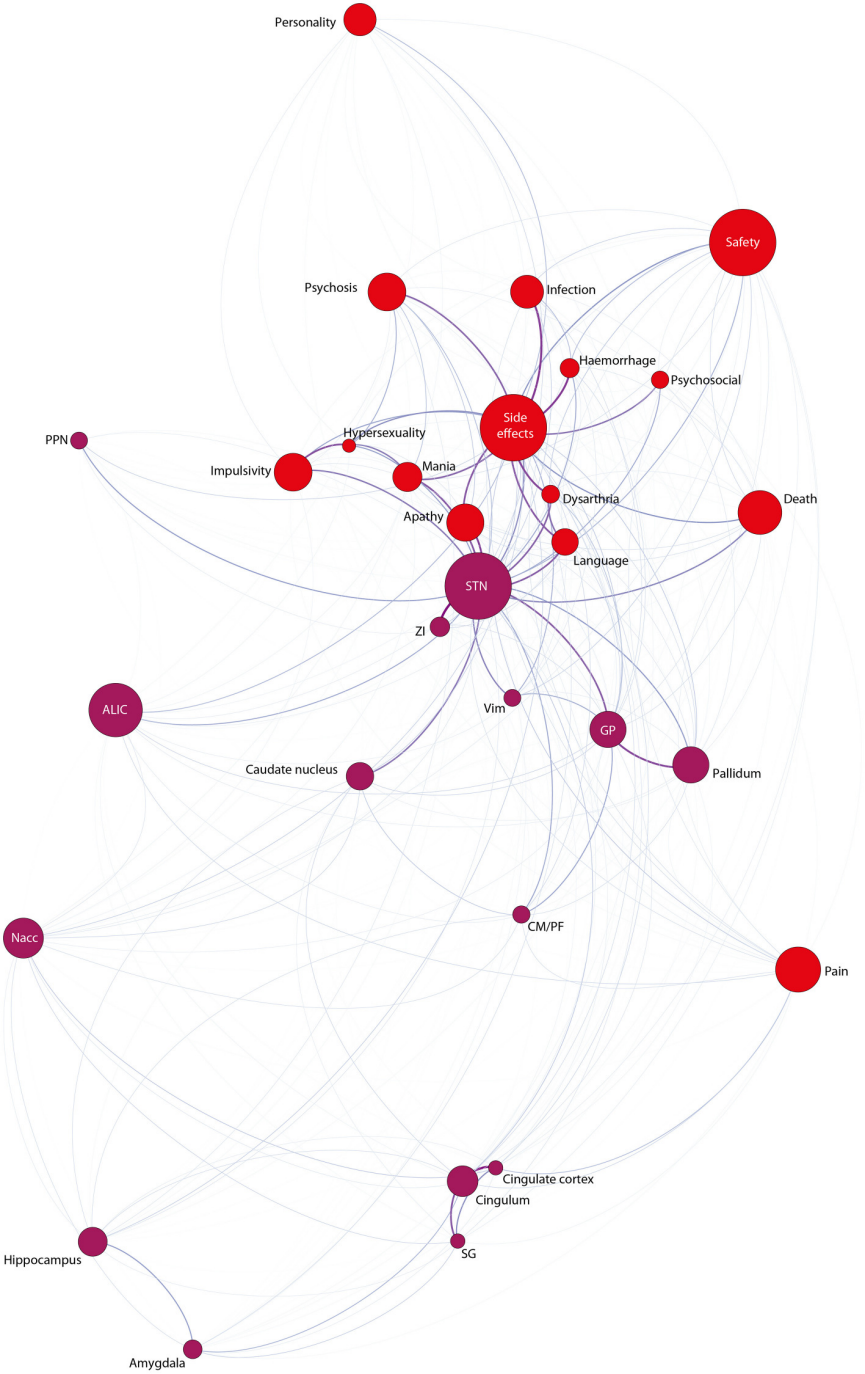
**Network-Analysis for the issues negative effects (red nodes) and anatomical targets (purple nodes)**.

Finally, the strongest co-occurrence connections between the issues side-effects and anatomical targets (Figure 7) yielded the following results: The STN is most often discussed with apathy, mania, speech, dysarthria, impulsivity, death, and hypersexuality. We found no robust co-occurrence between neuroanatomical targets relevant for the treatment of psychiatric disorders and concrete side-effects (such as infection and the like). Also no marked co-occurrence of side-effects and anatomical targets other than the STN were observed.

#### Additive relationships

Additively, we were interested in potential connections between topics from economic issues, technological issues, and positive effects.

The topic industry is most often discussed with PD, imaging methods, safety, and ET. Interestingly, terms like “costs,” “economic,” “commercial” and the like are markedly linked to the indication PD solely. The issue positive effects including the topics alleviation, relief, and remission are most often associated with PD, pain, and dopamine. The terms “quality of life” and “wellbeing” on the other hand are most often connected to PD, side-effect-related terms, psychosocial, and apathy while most prominent connections with regard to the topic enhance(ment) are PD, hardware, and STN. In particular, the strong connection between QoL and psychosocial is important because it may highlight an increased interest in psychosocial issues in the context of QoL. Finally the topic program is most often discussed with PD and STN whereas the topic of new-devices (terms: “closed loop” and “responsive neurostimulation system”) is most often connected to PD and epilepsy.

When integrating how methods other than DBS are discussed within the DBS-literature, one finds the following outcomes:

Concerning indications, PD is most often connected to subthalamotomy, dopamine, and pallidotomy. OCD on the other hand is robustly connected to capsulotomy and to a minor degree to cingulotomy whereas ET is mentioned most often in combination with thalamotomy. Finally, MDD is most often discussed with ECT, cingulotomy and still quite often with tDCS whereas epilepsy is most often discussed with VNS.

Regarding side-effects, dopamine is apparently most often discussed in combination with hypersexuality, impulsivity, psychosis, apathy, and dysarthria. Psychosurgery on the other hand, is mostly discussed in the context of cingulotomy whereas pain (here probably meant as indication) most often with SCS.

#### Associations with the topic “ethic”

**Figure 8:**
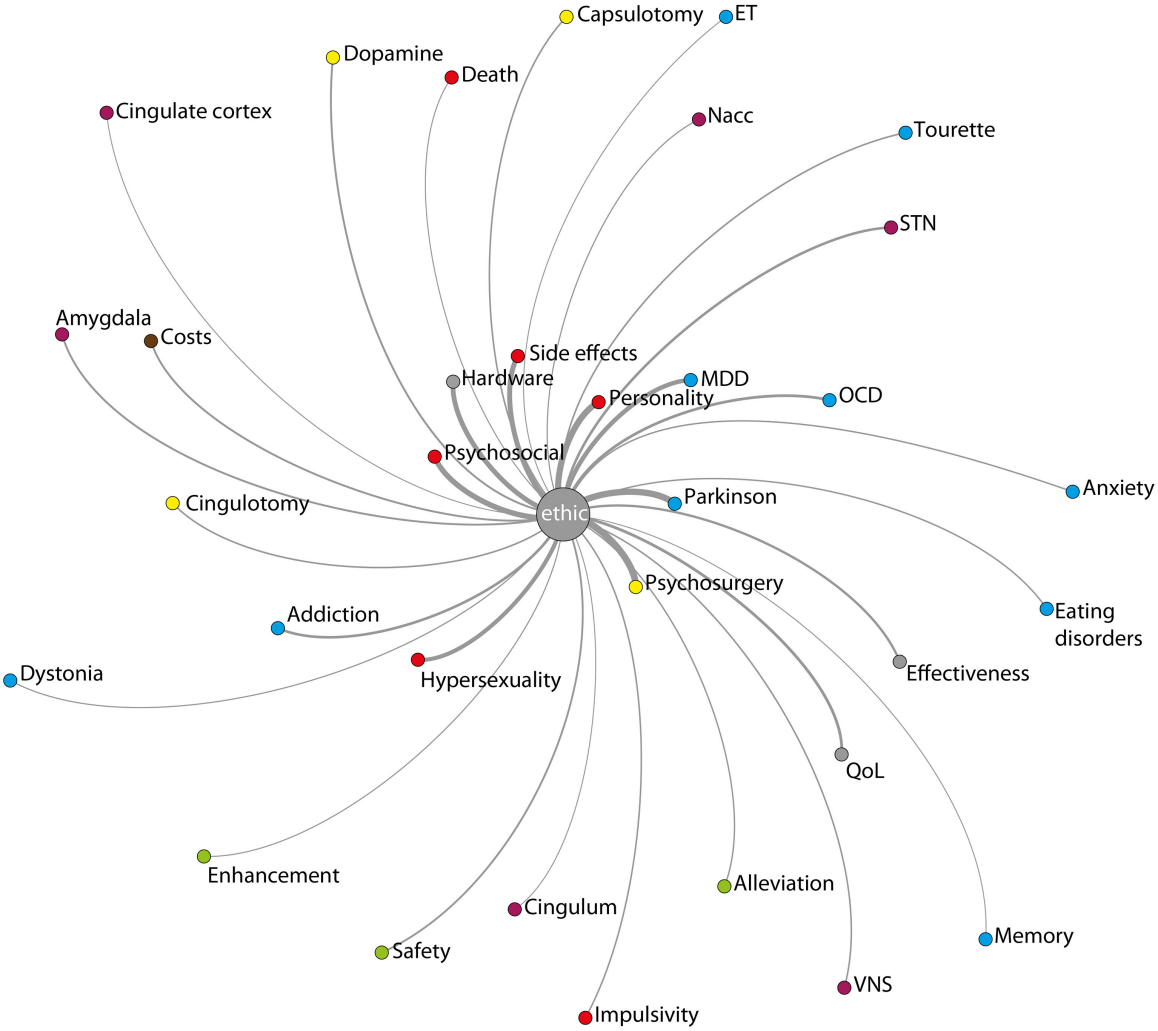
**Interrelation of the topic ethics to all other topics with at least 5% co-occurrence (for color code see caption of Figure 4)**.

There is a rich discussion which deals with ethical aspects in the context of DBS. Ranging from personality changes and side-effects (Christen et al., 2012) to topics taking up the debate of human enhancement (Synofzik and Schlaepfer, 2008; Schermer, 2013) and research ethics (Fins et al., 2011), the discussion is clearly multifaceted. Hence, ethical questions are a constant topic of debate. Therefore, we were interested in how ethics-terms interrelate with other terms of the text corpus. We therefore investigated the co-occurrence of the topic ethics with all other topics (see Figure 8). Apart from the rare topic psychosurgery (present in only 52 texts) and the very frequent topic PD (present in 3544 texts) which yielded the strongest co-occurrences, the ethics-topic is most often linked to personality, psychosocial, side effect, hardware, MDD, and hypersexuality. Of note is the very rare connection between ethical issues to the GP (in only 3% of the total possible co-occurrences) compared to the STN (in 14% of the total possible co-occurrences).

## Discussion and conclusion

We will first outline some of the findings which underpin the validity of our approach directly followed by discussing findings acquired from the trend analysis over time and then guide the discussion toward the graph analysis based on BC and co-occurrence. Finally, we will outline pertinent ethical questions.

### Findings corroborating the validity of our approach

We detected a multitude of findings which underpin the validity of our approach, some of which we highlight in the following. Chronic pain for example, is a well-described indication suitable for spinal cord stimulation (Wolter, 2014). The findings including indications and lesion methods also confirm known connections as evidenced in the case of ECT, a well-known therapeutic option for the treatment of MDD, besides others. The relationship of epilepsy and closed-loop systems might also underpin the robustness of our methodological approach by bearing in mind that epilepsy characterizes a promising indication for the application of closed loop devices (Armstrong et al., 2013; Krook-Magnuson et al., 2013; Paz et al., 2013; Nagaraj et al., 2015). Regarding anatomical targets and side-effects, we highlight a distinct connectivity between the STN and impulsivity which has been described elsewhere (Zavala et al., 2015). Regarding the detected intracategorial connection between impulsivity and hypersexuality, a recent publication confirms a tight connection between the two topics (Kor et al., 2013). Hence, a multitude of identified co-occurrences incorporating the different issues such as indications and corresponding lesion methods, or indications and anatomical targets among others, serve as validation of our method.

### Trend analysis

When analyzing the data set including DBS-indications and relevant anatomical targets, one can identify a clear shift away from motor-related neuroanatomical targets (GP, Vim, STN) (even though numerically still predominant) toward an emphasis on anatomical targets which are especially relevant in psychiatric indications (Nacc, amygdala, hippocampus, SC) (see Figures 3A,B). Such a tendency is indicative for the broadening of the therapeutic spectrum of DBS. A multitude of scientific publications highlighted this circumstance already (e.g., Hariz, 2012; Hariz et al., 2013; Christen et al., 2014, to name a few). Even though we cannot make a qualitative statement about how such topics are being discussed (i.e., the discussion may be framed in a supportive, critical, or neutral way), the identified increase may represent the seen utility in DBS for the treatment of psychiatric indications. Questions about suitability of such complex disorders for the therapeutic application of DBS and the difficult search of anatomical loci for the treatment of such indications (e.g., for MDD (among others): Nacc, ALIC, and SG) may also be indicative for such an increase. Additionally, the data highlights no standard locus for the treatment of MDD (e.g., Hariz et al., 2013 for the unspecific use of neurostimulation targets in the context of MDD, TS, and OCD; or Da Cunha et al., 2015; Kocabicak et al., 2015). Finally, the trend analysis incorporating negative effects (data not shown) completes the picture; while motor side effects are less frequently discussed, complex issues such as personality are increasingly the topic of the current debate. In the context of psychiatric disorders, such phenomena seem to be prevalent to a greater extent.

The trend analysis involving lesion based therapy approaches revealed that lesion approaches recede in the context of motor-related disorders and are quite stable in the case of psychiatric disorders. This might demonstrate—and under the assumption that medication based therapy is still the most frequently used therapeutic approach—that (1) lesions are still considered to be an effective and reliable means for patients refractory to drug-therapy and (2) that DBS was not (yet) able to replace lesion-based therapy approaches in psychiatry. When talking about an observed decline of the discussion of topics related to lesion approaches, one has to emphatically point out that this reflects how lesions are being discussed within the DBS-literature only. This means that we are limited in our interpretation of observations related to lesion methods and look with a narrow “DBS-perspective” on relationships which are discussed in these articles. Moreover, lesion approaches are still considered therapeutic competitors and as such might receive little attention. We have outlined elsewhere (Christen et al., 2014, but see also Müller et al., 2015) the importance of ensuring alternative therapeutic approaches which of course would not quantitatively carry much weight when extracting abstracts from numerous DBS-publications.

### Network analysis

#### Thematic structure of DBS publications

Our results suggest that the topics PD, side-effect, hardware, safety, and effectiveness play a conducive role within the DBS literature and this to a greater degree than other terms because the relation of their influence to the total number of connections was calculated to be highest (reflected in BC). One could say that the backbone of any publication in the context of DBS is composed of issues about safety, side-effects, effectiveness, hardware, and PD. These junctions act as mediators within the discursive field of the textual graph. The broader backbone of a publication in the field of DBS can be inferred to be generated through the mentioning of the remaining major topics: relief, MDD, imaging methods, dopamine, QoL, STN, dystonia, OCD, ALIC, pain, enhance(ment), epilepsy, death, ET, and TMS. This furthermore means that by analyzing the top 20 terms and by allocating those to the major topics, it is evident that indications are most numerously represented (*n* = 7), followed by negative effects (*n* = 4), methods (*n* = 3), and anatomical targets (*n* = 2). As the discussion in the DBS literature is shifting toward new and more specific questions, specific anatomical targets tend to be less often associated with more general topics. The strong representation of indications again reflects the trend of broadening the therapeutic spectrum in the context of DBS.

#### Personality and psychosocial issues in DBS publications

The growing discussion about personality (trend analysis) is not yet reflected in BC because the topic personality shows one of the lowest BC-values. This can be explained by referring to the low frequency of the topic itself within the whole text corpus and may likely change as such issues have to be addressed in the context of measuring pre-post-effects in the case of psychiatric neurostimulation. The circumstance of personality and psychosocial issues receiving low BC may indicate that their associated concepts represent genuinely vague and difficult variables and consequently are not utterly useful for clinical research. As validated instruments to objectively and qualitatively measure changes in the personality and the psychosocial dimension are often missing or criticized for not accurately measuring the topic under investigation, such much needed concepts cannot easily enter clinical research (Dimitrov and Rumrill, 2003). The fact that psychiatric indications are increasingly being addressed by means of brain-stimulation, the need for the accurate and thorough observation and measure of psychosocial and personality-related issues (and also in the context of movement and other disorders, Pham et al., 2015) is obviously most important. Therefore, with more accurate insights into the neuronal circuitries exerting maladaptive effects on many disorders despite high complexity and limited means of investigation (Rossi et al., 2015) and the eagerness to evaluate results beyond short-term quality of life (Ooms et al., 2014), indications for DBS should get more individually tailored (Galati and Stefani, 2015). Additively, the accurate and longitudinal measure of psychosocial issues has already been proposed also in the context of movement disorders (Schüpbach and Agid, 2008). Combined with the fact that the topic QoL is robustly connected to the topic psychosocial, as well as the fact that a limited number of scales for the measurement of personality- and psychosocial-related issues do exist, the introduction of such instruments combined with the eagerness to improve such instruments, is greatly needed.

#### Economic issues

The restriction of the discussion incorporating economic issues to PD only, also poses questions. Given the increase of psychiatric indications, economic considerations should be in place and extended toward other indications in order to adequately address socio-economic issues. The often observed co-occurrence between the topic economic and ethic further emphasizes this point.

#### Centralities of neuroanatomical targets and their implications

The BC-values of neuroanatomical targets indicate a higher one for ALIC than for GP and a higher one for Nacc than for Vim. This may serve as another evidence for the growing importance of neuropsychiatric topics in the literature of DBS. However, this sequence changes when conducting Page-rank analysis: highest values are accredited to the STN, GP, ALIC, Vim, and Nacc. Since Page-rank puts an emphasis on the number of connections (e.g., “links”), the traditional motor targets STN and GP would be listed before ALIC within a given search result. Given the fact that GP and Vim represent historically older topics in DBS and based on the higher Page-rank values, the two are more densely linked with other topics. However, ALIC and Nacc already are more central concepts within the DBS-literature, presumably acting as mediators of information to a greater extent than GP and Vim.

#### MDD and alternative therapeutic approaches

The execution of a Page-rank analysis also changes the sequence of the most important topics: PD clearly is attributed the highest value and MDD makes it into the “top 5.” In sum, one can state that depression is the most discussed psychiatric indication in the DBS literature. In light of MDD's importance within the DBS literature, it is from a bioethical point-of-view important to emphasize that this indication has not yet received approval from the U.S. Food and Drug Administration (FDA) as a standard therapeutic treatment. Patients therefore should be well-informed about the ongoing search of optimal neuroanatomical targets, the challenging support without standardized guidelines of patients along the whole treatment and beyond as well as the complexities associated with the appropriate conduct of clinical trials (Jimenez-Shahed, 2015) and the vulnerability of patients (Bell et al., 2014).

When looking specifically at alternative therapeutic approaches such as SCS, tDCS, ECT, and VNS, it becomes obvious that they receive especially low BC. This circumstance can be explained by highlighting that we specifically selected scientific publications involving DBS. Presumably, those publications have a low interest in advocating for alternative therapeutic approaches. Furthermore, the co-occurrence analysis shows that within the DBS-literature, ablative therapeutic approaches such as pallidotomy, subthalamotomy, and thalamotomy are most frequently discussed in combination with terms from the topic side effect. While ablative therapeutic strategies appear to a greater degree to be negatively connoted, the latter also symbolize the still most direct competitors to DBS, as already outlined within the trend-analysis. Interestingly, the lesional approaches (pallidotomy, subthalamotomy, and thalamotomy) receive dramatically more weight (in the middle field) when performing Page-rank (among lowest BC). In analogy of the previous hypothesis, lesion approaches might be underestimated if incorporating BC only and seem to be interlinked to a greater extent.

#### Discussion of issue-comparisons

The specific comparison involving indications and side-effects indicates that the description of side effects is clearly dominated by the ones closely associated with PD, the indication for which most publications exist (*n* = 3544, followed by essential tremor, *n* = 901). This does not mean that PD is the DBS indication where most side effects occur, but that side effects are most frequently described in the context of PD. As outlined above, the strong connection between PD and hypersexuality only reflects that within the few papers dealing with hypersexuality, the term “parkinson” is almost always present. Since the topic hypersexuality is very infrequent, this result has to be taken with caution. On the other hand, one also has to take into account that our data consist of abstracts only, i.e., terms need to have some importance within a paper in order to appear in the abstract. Side effects of other indications, especially neuropsychiatric, are to a far lesser degree discussed. Depression for example co-occurs only to a minimal extent with personality, death, and psychosocial issues. As highlighted previously, side effects in the context of psychiatric disorders are expected to be (1) much harder to be identified and (2) still have to be published as such newer indications have only recently been added to the therapeutic spectrum. OCD with its unconventional entry into the therapeutic landscape via a humanitarian device exemption (Fins et al., 2011) is also quite rarely discussed in the context of concrete side effects [probability of co-occurrence: hypersexuality (11%), impulsivity (8%), and infection (5%)]. Again, there is a duty to longitudinally follow patients in order to constantly monitor potential side-effects, besides the great need for introducing new measures in order to fully capture potential changes also in the psychosocial/psychiatric domain (Lilleeng et al., 2015).

The comparison involving indications and neuroanatomical targets highlights a further interesting result: apart from being discussed most often with the STN, PD is also quite strongly connected to the PPN, even more than to the GP—the other standard target for stimulation apart from the STN. PPN-stimulation was initially promoted for the treatment of balance impairments as well as refractory gait freezing and has been shown to be used as surgical target relatively unspecifically (“the PPN-area”) and this despite largely unknown clinical usefulness (Hariz et al., 2013). The high centrality of the PPN together with such a critical stance toward its usefulness and lack of clinical evidence further corroborates an apparent tension.

Finally, when comparing side-effects and anatomical targets, we observe again a dominant description of side-effects in combination with the STN—one of the most widely used anatomical targets for the treatment of movement disorders. The most frequent co-occurrence of the GP is its connection to apathy, but this happens only in 7.5% of cases in which a possible co-occurrence is possible. This, potentially driven by stimulation of ventral and medial subterritories of the STN (for STN-subterritories see Tremblay et al., 2015; for actions beyond motor control see Zavala et al., 2015), reflects a described dominance of side-effects in the context of STN rather than other (e.g., GPi) DBS targets. Moreover, this could indicate that side-effects emanating from predominantly ventral STN-stimulation have overshadowed the description of side-effects of other anatomical targets such as the GP. Alternatively, the STN may be intrinsically more prone to (behavioral or affective) side effects due to its circumscribed connectivity to limbic areas. There is evidence for a clearer separation of motor and non-motor functions in the GPi compared to the STN (Wichmann and DeLong, 2011; Da Cunha et al., 2015). Additionally, ALIC, an anatomical target with marked BC, is rarely found in combination with specific side effects. This imbalance may be problematic or may be the result of the already mentioned problem of capturing side-effects in the context of psychiatric disorders. However, if DBS for the neuropsychiatric domain further expands, the accurate description combined with the nuanced measurement of psychological changes poses a bioethical obligation and responsibility for any researcher involved. It might be an interesting endeavor to once try to capture the implicit perception of professionals in the field of DBS regarding such issues. The numerical imbalance of how e.g., anatomical targets are discussed in relation to side-effects together with the concrete framing of such issues within the most often read articles in the literature supposedly induce unconscious preferences which do not necessarily display the situation in an accurate way based on (pre-)clinical evidence.

### Bioethical issues

As the field of bioethics is comparably subjected to a vast increase of publications, quantitative methods for obtaining a better understanding might be as important as for neuroscience. Such an approach is moreover suitable for identifying potential mismatches between what is currently being discussed and what might be important ethical topics which are less tangible or more vulnerable for being overlooked.

#### Motor-targets and their connection to ethics

Apparently, ethical issues are to a greater extent discussed in combination with the STN rather than the GP. Is the STN therefore more thoroughly described in terms of its stimulation-based ethical consequences or is there evidence that the STN intrinsically harbors to a greater extent problematic ethical issues including the potential of inducing undesired effects? The debate of which target is most appropriate (mostly including STN and GP, Follett et al., 2010; Krack and Hariz, 2010; Odekerken et al., 2013) is an old one. But since currently, there is evidence for a statistical chance selection rather than one based on (patho)-physiological evidence for either receiving STN or GP stimulation (Christen et al., 2014; Gilbert, 2014) [besides clinical considerations such as envisaging drug reduction (STN) or preexistence of cognitive symptoms (GPi) (Da Cunha et al., 2015)], this difference might be an important one. As long as there is no proven display regarding superiority in terms of therapeutic action, there might be a duty to investigate ethical issues to a similar extent for all nuclei used for stimulation.

#### Depression and its connection to ethics

Another interesting finding is an observed imbalance reflected in a strong connection between the issue which incorporates the topic ethics on the one hand and MDD on the other and a substantially weak connection of the former topic and the one involving the most often used anatomical targets in the context of depression. Whenever ethical issues are being discussed, MDD is most often also discussed. However, the factual co-occurrence dramatically decreases in the case of neuropsychiatric anatomical targets: ethical issues co-occur only in 6.5% of cases when discussing the Nacc, in 3.5% when discussing the ALIC and in 1.75% when discussing the SG. This apparent dissociation between indication and anatomical target is questionable and more pronounced as in the case of PD and STN. As DBS has not faced a comparably long history regarding randomized controlled clinical trials for psychiatric disorders, studies have to be continued in order to identify which nucleus (or nuclei) shows greatest potential for the treatment of MDD and other neuropsychiatric disorders but also to identify which nucleus might be especially vulnerable for (behavioral) side-effects, psychosocial maladjustments and consequently ethical issues (e.g., non-maleficence).

#### Hardware related issues

As evidenced in some publications (Kondziolka et al., 2001; Okun et al., 2005; Fins, 2009) hardware related complications do impose ethical challenges. This is also backed by our results highlighting an apparent tension between hardware and ethical issues. Concomitantly, our data set indicates a continuous decrease of the discussion of hardware related topics (evidenced in the trend analysis; data not shown) but also particularly high BC. The strong link to ethical issues, apart from the mere description of hardware-related side-effects, might be evaluated as unintuitive. However, the data suggests that hardware related issues in the context of social, ethical and moral questions apparently have already been a topic of debate (e.g., Hilimire et al., 2015; Fumagalli et al., 2015). Generally, the topic of ethical and social implications of technological devices is certainly an important one which, e.g., in the context of emerging closed-loop devices, will nourish further discussions in the future. Therefore, closely investigating ethical, social, and clinical aspects of the follow up process longitudinally, including e.g., the often challenging postoperative phase for precise DBS parameter adjustments (Ineichen et al., 2014) as well as fiscal and legal aspects of hardware replacement apart from ethical issues specifically in the context of hardware is important. In parallel, our result may emphasize not only a duty to investigate hardware related ethical issues which transcend merely and well-known technical problems (Christen et al., 2014) thoroughly, but also that ethical duties already instantiated also apply to engineers which represent key players and which are well-positioned to support the deployment of innovative hardware in order to diminish the burden of patients (Fins, 2009). In the general DBS review literature, hardware-related issues such as the ones attached to recording devices and the related implications for patients' autonomy and responsibility but also the potential use and abuse of such recorded signals in connection with privacy issues, the dependency on device manufacturers (Underwood, 2015) and conflicting interests (Clausen, 2011), the long-term risk of living with implanted hardware (Farris et al., 2008) apart from psychological issues have in comparison to surgical complications probably received less attention. The fact that hardware-related issues receive dramatically more weight in the context of ethical challenges than impulsivity, concrete side-effects and death/suicide is certainly surprising and needs further analysis. Whether this means that hardware-related issues are already sufficiently discussed in an ethical and social context or need further exploration has to be identified by a qualitative in-depth analysis.

Although DBS has alleviated patients suffering tremendously, many obstacles still remain. Recently, the development of innovative neuromodulation exemplified by current steering (Martens et al., 2011; Hariz, 2014b), adaptive DBS (Little et al., 2014) but also the potential deployment of closed-loop devices (e.g., Rosin et al., 2011; Grahn et al., 2014; Williams, 2015) have increasingly gained weight within the discussion of DBS. In the meanwhile, magnetothermal neuromodulation in translational models (Chen et al., 2015) shows potential to increase our knowledge of neuronal microcircuitries (Temel and Jahanshahi, 2015). Apart from technological as well as biological hurdles (e.g., identification of true biomarkers) also ethical issues might arise. As our data highlights a tremendously weak co-occurrence of the topics ethics and closed-loop, it might be time to think about how emerging closed-loop devices may affect already instantiated guidelines and what differences as well as implications might be identified both from a theoretical (i.e., philosophical) but also practical (i.e., what it would mean for patients) perspective.

### Limitations

This study, of course, incorporates some limitations. First of all, as in any quantitative text-network approach, we are unable to make qualitative statements. This however, is within the nature of a heuristics approach. Additionally, various topics might in fact be used in a different context than used as a basis for interpretation within this study. For example, the neuroanatomical targets may in fact very well also be mentioned without referring to a target for stimulation. For example, the STN may be described within a DBS-publication not as a target for stimulation but within the context of hyper-reactivity in the case of hemiballism. Due to the fact that we have limited our analysis to abstracts, we assume this scenario to be quite infrequent and would hypothesize such a wording to be included in a general introduction rather than within an abstract. Moreover, the Web of Science database is associated with a language bias. As papers emphasizing psychosocial and philosophical issues are often published in other languages, they are likely to be underrepresented in our sample. Finally, topics which incorporated multiple terms, of course, inevitably have a greater probability of co-occurrence.

### Outlook

The proposed analysis is by no means complete and has no prerogative of accuracy. It is one additional possibility to read any text in order to gain new insights about its structure and hidden messages, suitable to deal with a large number of texts. We are of the opinion that applying network approaches, visualization techniques and graph theory to a text corpus might be an innovative and promising alternative which entails fruitful and worth considering aspects. The final interpretation of the data, once visualized as a graph, is certainly open for discussions and by no means definitive.

Hariz recently wrote in his book chapter “The literature revisited” that “serendipitous discoveries and advances in functional imaging are providing ‘new’ brain targets for an increasing number of pathologies, and the corollary is an exponentially growing literature on DBS, such that it is simply impossible to keep track of all papers and books appearing on this subject.” He then goes on by stating “While most of the literature constitutes an invaluable wealth of knowledge, a small but important part gives rise to serious concern and needs to be revisited and discussed” (Hariz, 2014a). By using a quantitative network approach, we tackled this issue from another perspective and tried to identify potentially hidden and underrepresented issues which might be relevant for further discussions and future research.

## Author contributions

CI extracted the data, MC generated the final set, CI and MC analyzed the data and wrote the paper. CI furthermore confirms that he has final responsibility for the decision to submit for publication.

### Conflict of interest statement

The authors declare that the research was conducted in the absence of any commercial or financial relationships that could be construed as a potential conflict of interest.

## Footnotes

^1^WoS, Thomson Reuters, access through https://webofknowledge.com/

^2^Available at: https://code.google.com/p/stop-words/

^3^Available at: http://www.lexiconista.com/datasets/lemmatization/

^4^The text length distribution displayed a peak for very short texts (e.g., editorial material that is only present in the WoS database with its title). In the mean, there were 36 texts per bin, the standard deviation was 57. Thus, the distribution was cut where there were more than 36+57 = 93 texts per bin, which was the case for texts that contained 11 or less words).

^5^In some cases, we also replaced verbs or adjectives with nouns; e.g., “painful” was replaced with “pain,” because the number of words of one category was considerably lower compared to the number of words of the other category.

^6^Available at http://gephi.github.io/

## Abbreviations

ALIC: Anterior limb of internal capsule
BC: Betweenness Centrality
DBS: Deep Brain Stimulation
ECT: Electro-convulsive therapy
ET: Essential tremor
GP: Globus pallidus
GPi: Globus pallidus internal segment
MDD: Major depressive disorder
STN: Nucleus subthalamicus
Nacc: Nucleus accumbens
OCD: Obsessive-compulsive disorder
PD: Parkinson's disease
PPN: Pedunculo-pontine nucleus
QoL: Quality of life
SCS: Spinal cord stimulation
SG: Subgenual cingulate
TS: Tourette syndrome
tDCS: Transcranial direct current stimulation
VNS: Vagus nerve stimulation
Vim: Ventral intermediate nucleus
Zi: Zona incerta.
