# Supplementary material for: Analyzing 7000 texts on deep brain stimulation: what do they tell us?
Source: Front Integr Neurosci. 2015 Oct 26;9:52. doi: 10.3389/fnint.2015.00052 (PMC4620160; doi:10.3389/fnint.2015.00052)
Supplement: Supplementary file 1 [file Table1.DOCX]

***Supplementary Material***

**Analyzing 7000 texts on Deep Brain Stimulation: what do they tell us?**

**Christian Ineichen^1,2^* and Markus Christen^1,3^**

^1^Institute of Biomedical Ethics and History of Medicine, University of Zurich, Zurich, Switzerland.

^2^Preclinical Laboratory for Translational Research into Affective Disorders, Clinic for Affective Disorders and General Psychiatry, Psychiatric University Hospital Zurich, Zurich, Switzerland

^3^University Research Priority Program Ethics, University of Zurich, Zurich, Switzerland

*** Correspondence:** Christian Ineichen, [christian.ineichen@uzh.ch](mailto:christian.ineichen@uzh.ch)

1. **Supplementary Data**
2. **Supplementary Figures and Tables**

## Supplementary Tables

**Table 1: BC- and Page-rank values of all topics (except ethic)**

| **Label** | **Betweenness Centrality** | **PageRank** |
| --- | --- | --- |
| safety | 27,247 | 0,0175 |
| parkinson | 27,247 | 0,0583 |
| effectiveness | 27,247 | 0,0293 |
| sideeffect | 27,247 | 0,0367 |
| hardware | 27,247 | 0,0446 |
| alleviation | 26,106 | 0,0168 |
| depression | 25,638 | 0,0294 |
| computertomography | 24,588 | 0,0205 |
| dopamine | 24,363 | 0,0261 |
| qualityoflife | 22,203 | 0,0135 |
| subthalamicus | 21,134 | 0,0346 |
| dystonia | 19,714 | 0,0172 |
| obsessivecompulsive | 19,272 | 0,0178 |
| alic | 19,136 | 0,0116 |
| pain | 17,950 | 0,0138 |
| enhancement | 17,704 | 0,0106 |
| epilepsy | 17,643 | 0,0135 |
| death | 17,268 | 0,0108 |
| tremor | 17,179 | 0,0232 |
| transcranialmagneticstimulation | 15,225 | 0,0122 |
| anxiety | 14,099 | 0,0128 |
| program | 12,478 | 0,0099 |
| globuspallidus | 12,303 | 0,0178 |
| ablation | 11,861 | 0,0130 |
| dyskinesia | 11,543 | 0,0158 |
| pallidum | 11,157 | 0,0125 |
| infection | 10,455 | 0,0098 |
| memory | 10,273 | 0,0104 |
| accumbens | 9,895 | 0,0098 |
| psychosis | 9,837 | 0,0123 |
| tourette | 9,714 | 0,0106 |
| addiction | 9,564 | 0,0106 |
| bradykinesia | 7,753 | 0,0115 |
| economic | 7,415 | 0,0088 |
| cingulum | 7,034 | 0,0119 |
| hippocampus | 6,671 | 0,0095 |
| sclerosis | 6,319 | 0,0105 |
| apathy | 6,072 | 0,0117 |
| mania | 5,898 | 0,0115 |
| speech | 5,753 | 0,0105 |
| personality | 5,424 | 0,0078 |
| haemorrhage | 5,049 | 0,0103 |
| psychosurgery | 4,826 | 0,0088 |
| radiosurgery | 4,706 | 0,0084 |
| headache | 4,528 | 0,0074 |
| vagusnervestimulation | 4,377 | 0,0099 |
| vim | 4,178 | 0,0113 |
| caudatenucleus | 3,982 | 0,0096 |
| thalamotomy | 3,959 | 0,0122 |
| impulsivity | 3,912 | 0,0097 |
| closedloop | 3,545 | 0,0081 |
| alzheimer | 3,409 | 0,0093 |
| pallidotomy | 3,356 | 0,0123 |
| amygdala | 3,262 | 0,0081 |
| electroconvulsivetherapy | 3,246 | 0,0097 |
| psychosocial | 2,984 | 0,0092 |
| subgenual | 2,732 | 0,0095 |
| dysarthria | 2,632 | 0,0118 |
| centromedianparafascicularcomplex | 2,581 | 0,0097 |
| eatingdisorders | 2,523 | 0,0096 |
| zonaincerta | 2,295 | 0,0091 |
| chorea | 2,229 | 0,0088 |
| pedunculopontinenucleus | 2,186 | 0,0067 |
| cingulatecortex | 1,859 | 0,0098 |
| industry | 1,612 | 0,0076 |
| ataxia | 1,434 | 0,0084 |
| capsulotomy | 1,323 | 0,0094 |
| transcranialdirectcurrentstimulation | 1,297 | 0,0092 |
| schizophrenia | 1,168 | 0,0084 |
| cingulotomy | 0,942 | 0,0101 |
| subthalamotomy | 0,456 | 0,0120 |
| hypersexuality | 0,346 | 0,0107 |
| spinalcordstimulation | 0,190 | 0,0077 |

**Table 2: Listed co-occurrences** > 0.3

| **Node1** | **Node2** | **Weight** |
| --- | --- | --- |
| hypersexuality | parkinson | 0,947 |
| parkinson | bradykinesia | 0,938 |
| subthalamotomy | parkinson | 0,929 |
| apathy | parkinson | 0,906 |
| parkinson | dyskinesia | 0,896 |
| dopamine | parkinson | 0,892 |
| parkinson | subthalamicus | 0,877 |
| hardware | industry | 0,873 |
| parkinson | pedunculopontinenucleus | 0,850 |
| tremor | vim | 0,838 |
| pallidotomy | parkinson | 0,807 |
| capsulotomy | obsessivecompulsive | 0,800 |
| depression | subgenual | 0,789 |
| subthalamotomy | subthalamicus | 0,786 |
| parkinson | mania | 0,745 |
| impulsivity | parkinson | 0,741 |
| hypersexuality | dopamine | 0,737 |
| speech | parkinson | 0,725 |
| zonaincerta | subthalamicus | 0,699 |
| parkinson | zonaincerta | 0,699 |
| apathy | depression | 0,698 |
| sclerosis | tremor | 0,697 |
| dopamine | dyskinesia | 0,695 |
| cingulum | cingulatecortex | 0,684 |
| thalamotomy | tremor | 0,683 |
| hardware | haemorrhage | 0,646 |
| electroconvulsivetherapy | depression | 0,646 |
| dysarthria | parkinson | 0,643 |
| parkinson | alzheimer | 0,641 |
| pain | spinalcordstimulation | 0,640 |
| qualityoflife | parkinson | 0,638 |
| depression | anxiety | 0,630 |
| sideeffect | parkinson | 0,630 |
| parkinson | tremor | 0,629 |
| psychosocial | parkinson | 0,628 |
| closedloop | hardware | 0,621 |
| hardware | infection | 0,618 |
| transcranialmagneticstimulation | transcranialdirectcurrentstimulation | 0,615 |
| program | hardware | 0,598 |
| bradykinesia | subthalamicus | 0,594 |
| infection | sideeffect | 0,590 |
| program | parkinson | 0,586 |
| haemorrhage | sideeffect | 0,586 |
| death | parkinson | 0,581 |
| parkinson | globuspallidus | 0,569 |
| apathy | subthalamicus | 0,566 |
| cingulotomy | depression | 0,565 |
| impulsivity | dopamine | 0,565 |
| pallidum | globuspallidus | 0,555 |
| dysarthria | sideeffect | 0,554 |
| tremor | ataxia | 0,541 |
| psychosis | parkinson | 0,530 |
| impulsivity | hypersexuality | 0,526 |
| computertomography | cingulum | 0,526 |
| ablation | parkinson | 0,525 |
| cingulotomy | obsessivecompulsive | 0,522 |
| cingulotomy | capsulotomy | 0,522 |
| psychosurgery | cingulotomy | 0,522 |
| tremor | bradykinesia | 0,521 |
| vagusnervestimulation | epilepsy | 0,521 |
| dyskinesia | subthalamicus | 0,518 |
| parkinson | effectiveness | 0,517 |
| dopamine | subthalamicus | 0,511 |
| mania | subthalamicus | 0,510 |
| apathy | sideeffect | 0,509 |
| speech | sideeffect | 0,509 |
| subgenual | cingulum | 0,509 |
| alleviation | parkinson | 0,508 |
| parkinson | chorea | 0,500 |
| transcranialdirectcurrentstimulation | depression | 0,500 |
| psychosis | depression | 0,500 |
| subthalamicus | globuspallidus | 0,498 |
| dystonia | pallidum | 0,497 |
| speech | subthalamicus | 0,495 |
| parkinson | vim | 0,492 |
| electroconvulsivetherapy | transcranialmagneticstimulation | 0,492 |
| parkinson | pallidum | 0,486 |
| closedloop | parkinson | 0,485 |
| dysarthria | subthalamicus | 0,482 |
| psychosis | sideeffect | 0,480 |
| hardware | parkinson | 0,480 |
| parkinson | economic | 0,477 |
| depression | cingulatecortex | 0,474 |
| sideeffect | memory | 0,471 |
| mania | depression | 0,471 |
| sideeffect | mania | 0,471 |
| hardware | centromedianparafascicularcomplex | 0,467 |
| hardware | safety | 0,465 |
| parkinson | caudatenucleus | 0,464 |
| hardware | economic | 0,464 |
| hardware | computertomography | 0,463 |
| vagusnervestimulation | transcranialmagneticstimulation | 0,458 |
| thalamotomy | parkinson | 0,455 |
| computertomography | cingulatecortex | 0,447 |
| speech | dysarthria | 0,446 |
| safety | parkinson | 0,445 |
| pain | headache | 0,445 |
| sideeffect | dyskinesia | 0,442 |
| psychosurgery | obsessivecompulsive | 0,442 |
| psychosocial | sideeffect | 0,442 |
| tourette | obsessivecompulsive | 0,441 |
| enhancement | parkinson | 0,440 |
| computertomography | subgenual | 0,439 |
| parkinson | alic | 0,438 |
| vagusnervestimulation | depression | 0,438 |
| subthalamicus | caudatenucleus | 0,435 |
| pallidotomy | subthalamicus | 0,435 |
| dystonia | chorea | 0,431 |
| obsessivecompulsive | depression | 0,430 |
| hardware | pain | 0,429 |
| impulsivity | subthalamicus | 0,426 |
| sideeffect | qualityoflife | 0,425 |
| hardware | vim | 0,421 |
| hypersexuality | mania | 0,421 |
| personality | parkinson | 0,421 |
| computertomography | caudatenucleus | 0,420 |
| parkinson | memory | 0,414 |
| hardware | tremor | 0,413 |
| computertomography | parkinson | 0,406 |
| hardware | caudatenucleus | 0,406 |
| sideeffect | anxiety | 0,400 |
| psychosis | dopamine | 0,400 |
| epilepsy | hippocampus | 0,399 |
| sideeffect | pallidotomy | 0,398 |
| vim | subthalamicus | 0,396 |
| dopamine | bradykinesia | 0,396 |
| infection | parkinson | 0,396 |
| subthalamotomy | dyskinesia | 0,393 |
| dopamine | subthalamotomy | 0,393 |
| sideeffect | subthalamotomy | 0,393 |
| parkinson | depression | 0,390 |
| dystonia | globuspallidus | 0,390 |
| death | subthalamicus | 0,390 |
| obsessivecompulsive | eatingdisorders | 0,385 |
| hardware | epilepsy | 0,384 |
| sideeffect | ablation | 0,384 |
| sclerosis | parkinson | 0,382 |
| parkinson | dystonia | 0,381 |
| hardware | sideeffect | 0,380 |
| hardware | alleviation | 0,380 |
| closedloop | epilepsy | 0,379 |
| sideeffect | subthalamicus | 0,378 |
| depression | cingulum | 0,376 |
| parkinson | anxiety | 0,376 |
| hardware | death | 0,375 |
| hardware | dysarthria | 0,375 |
| psychosocial | qualityoflife | 0,372 |
| capsulotomy | depression | 0,371 |
| hardware | effectiveness | 0,370 |
| hardware | zonaincerta | 0,370 |
| hardware | bradykinesia | 0,370 |
| subgenual | cingulatecortex | 0,368 |
| hypersexuality | sideeffect | 0,368 |
| hardware | alic | 0,366 |
| psychosis | schizophrenia | 0,366 |
| hardware | subthalamicus | 0,362 |
| spinalcordstimulation | effectiveness | 0,360 |
| hardware | thalamotomy | 0,359 |
| apathy | dopamine | 0,358 |
| tremor | zonaincerta | 0,356 |
| hardware | dystonia | 0,356 |
| epilepsy | centromedianparafascicularcomplex | 0,356 |
| hardware | globuspallidus | 0,355 |
| hardware | vagusnervestimulation | 0,354 |
| pallidotomy | globuspallidus | 0,354 |
| haemorrhage | parkinson | 0,354 |
| death | sideeffect | 0,353 |
| sideeffect | safety | 0,351 |
| hardware | ablation | 0,350 |
| psychosurgery | depression | 0,346 |
| vagusnervestimulation | transcranialdirectcurrentstimulation | 0,346 |
| hardware | transcranialdirectcurrentstimulation | 0,346 |
| sideeffect | thalamotomy | 0,345 |
| program | subthalamicus | 0,345 |
| parkinson | industry | 0,342 |
| dopamine | pallidotomy | 0,342 |
| transcranialmagneticstimulation | depression | 0,341 |
| apathy | anxiety | 0,340 |
| obsessivecompulsive | accumbens | 0,337 |
| qualityoflife | subthalamicus | 0,337 |
| hardware | enhancement | 0,337 |
| computertomography | subthalamicus | 0,336 |
| alleviation | subthalamicus | 0,333 |
| subthalamicus | pallidum | 0,332 |
| hardware | hippocampus | 0,331 |
| safety | effectiveness | 0,331 |
| pallidotomy | dyskinesia | 0,329 |
| effectiveness | subthalamicus | 0,329 |
| sideeffect | bradykinesia | 0,328 |
| hardware | pallidum | 0,327 |
| eatingdisorders | effectiveness | 0,327 |
| parkinson | eatingdisorders | 0,327 |
| depression | addiction | 0,325 |
| parkinson | addiction | 0,325 |
| hardware | ataxia | 0,324 |
| hardware | transcranialmagneticstimulation | 0,324 |
| computertomography | radiosurgery | 0,324 |
| dysarthria | tremor | 0,321 |
| subthalamotomy | pallidotomy | 0,321 |
| tremor | subthalamicus | 0,321 |
| transcranialmagneticstimulation | spinalcordstimulation | 0,320 |
| hardware | spinalcordstimulation | 0,320 |
| sideeffect | dopamine | 0,318 |
| hippocampus | amygdala | 0,318 |
| subthalamicus | pedunculopontinenucleus | 0,317 |
| hypersexuality | subthalamicus | 0,316 |
| hardware | sclerosis | 0,315 |
| vagusnervestimulation | effectiveness | 0,313 |
| hardware | headache | 0,312 |
| hardware | pedunculopontinenucleus | 0,311 |
| thalamotomy | pallidotomy | 0,310 |
| alleviation | effectiveness | 0,310 |
| chorea | globuspallidus | 0,306 |
| impulsivity | sideeffect | 0,306 |
| effectiveness | vim | 0,305 |
| dysarthria | dopamine | 0,304 |
| depression | accumbens | 0,302 |

## Supplementary Figures

Supplementary Figure 1. (A) Text size distribution (number of words per texts) and (B) term frequency (frequency of terms in texts).
