# Supplementary figures and images for: Analyzing 7000 texts on deep brain stimulation: what do they tell us?
Source: Front Integr Neurosci. 2015 Oct 26;9:52. doi: 10.3389/fnint.2015.00052 (PMC4620160; doi:10.3389/fnint.2015.00052)

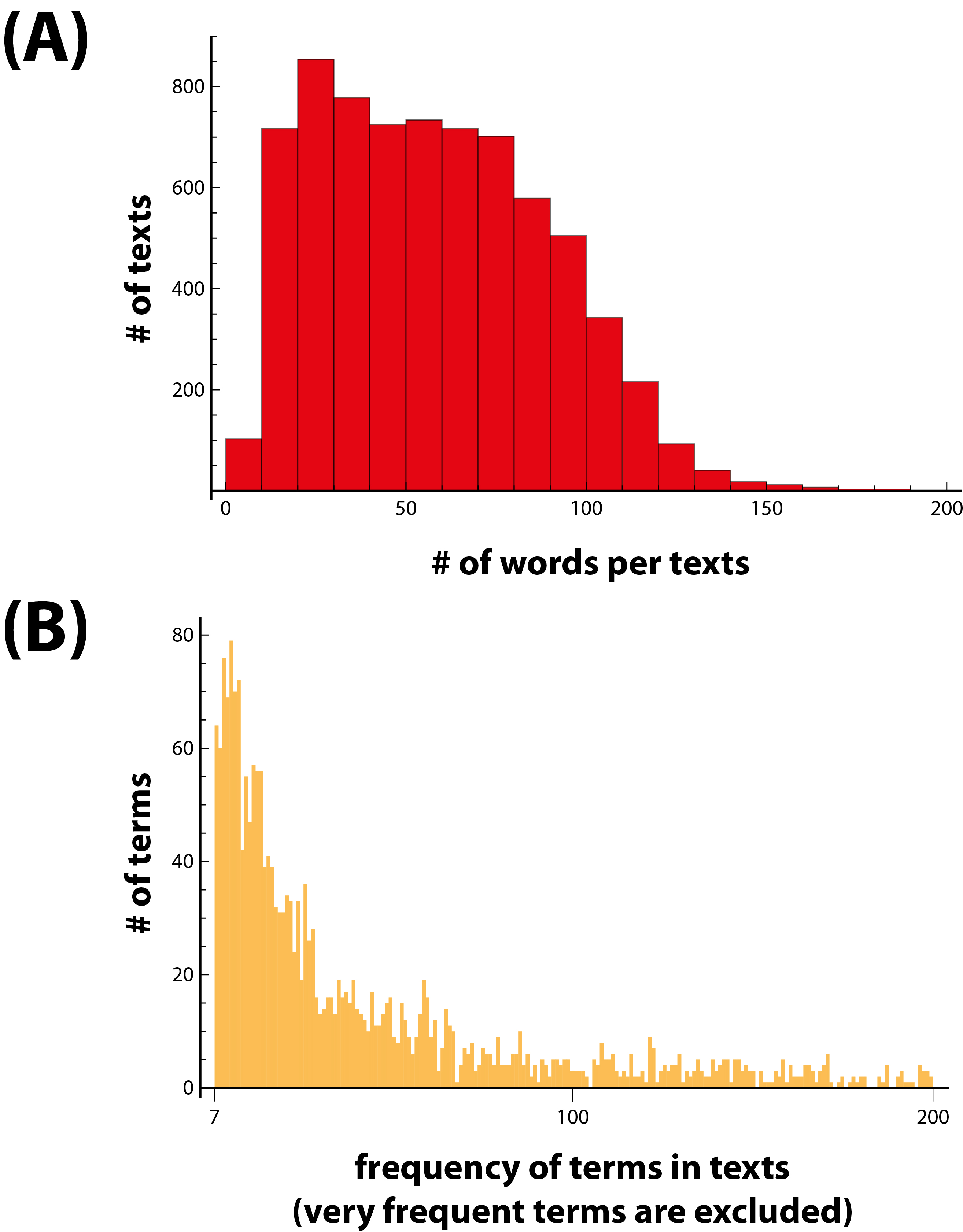

Supplement: Supplementary file 2 [file Image1.JPEG]
